# Intestinal Transcriptomes of Nematodes: Comparison of the Parasites *Ascaris suum* and *Haemonchus contortus* with the Free-living *Caenorhabditis elegans*

**DOI:** 10.1371/journal.pntd.0000269

**Published:** 2008-08-06

**Authors:** Yong Yin, John Martin, Sahar Abubucker, Alan L. Scott, James P. McCarter, Richard K. Wilson, Douglas P. Jasmer, Makedonka Mitreva

**Affiliations:** 1 Genome Sequencing Center, Department of Genetics, Washington University School of Medicine, St. Louis, Missouri, United States of America; 2 Department of Molecular Microbiology and Immunology, Johns Hopkins School of Public Health, Baltimore, Maryland, United States of America; 3 Divergence Inc., St. Louis, Missouri, United States of America; 4 Department of Veterinary Microbiology and Pathology, Washington State University, Pullman, Washington, United States of America; Queensland Institute of Medical Research, Australia

## Abstract

**Background:**

The nematode intestine is a major organ responsible for nutrient digestion and absorption; it is also involved in many other processes, such as reproduction, innate immunity, stress responses, and aging. The importance of the intestine as a target for the control of parasitic nematodes has been demonstrated. However, the lack of detailed knowledge on the molecular and cellular functions of the intestine and the level of its conservation across nematodes has impeded breakthroughs in this application.

**Methods and Findings:**

As part of an extensive effort to investigate various transcribed genomes from *Ascaris suum* and *Haemonchus contortus*, we generated a large collection of intestinal sequences from parasitic nematodes by identifying 3,121 *A. suum* and 1,755 *H. contortus* genes expressed in the adult intestine through the generation of expressed sequence tags. Cross-species comparisons to the intestine of the free-living *C. elegans* revealed substantial diversification in the adult intestinal transcriptomes among these species, suggesting lineage- or species-specific adaptations during nematode evolution. In contrast, significant conservation of the intestinal gene repertories was also evident, despite the evolutionary distance of ∼350 million years separating them. A group of 241 intestinal protein families (IntFam-241), each containing members from all three species, was identified based on sequence similarities. These conserved proteins accounted for ∼20% of the sampled intestinal transcriptomes from the three nematodes and are proposed to represent conserved core functions in the nematode intestine. Functional characterizations of the IntFam-241 suggested important roles in molecular functions such as protein kinases and proteases, and biological pathways of carbohydrate metabolism, energy metabolism, and translation. Conservation in the core protein families was further explored by extrapolating observable RNA interference phenotypes in *C. elegans* to their parasitic counterparts.

**Conclusions:**

Our study has provided novel insights into the nematode intestine and lays foundations for further comparative studies on biology, parasitism, and evolution within the phylum Nematoda.

## Author Summary

Biological properties of the nematode intestine warrant in-depth investigation, the results of which can be utilized in the control of parasitic nematodes that infect humans, livestock, and plants. Both the importance of intestinal antigens from *Haemonchus contortus* in immunity and the damage to *H. contortus* intestine by anthelmintic fenbendazole have highlighted the versatility of the intestine as an emerging target. However, biological information regarding fundamental intestinal cell functions and mechanisms is currently limited. Conserved intestinal genes across nematode pathogens could offer molecular targets for broad parasite control. Furthermore, qualitative and quantitative comparisons on intestinal gene expression among species and lineages can identify basic adaptations relative to a critical selective force, the nutrient acquisition. This study begins to identify intestinal cell characteristics that are conserved across representatives of two clades of nematodes (V and III) and further clarifies diversities that likely reflect species- or lineage-specific adaptations. Results consistent with functional data on digestive enzymes from *H. contortus* and RNAi in *Caenorhabditis elegans*, as examples, support the potential for the comparative genomics approach to produce practical applications. This study provides a platform on which extensive investigation of intestinal genes and a more comprehensive understanding of the Nematoda can be gained.

## Introduction

The intestine is one of the major organs in nematodes, creating a key surface at the intestinal apical membrane that interacts with the environment. While specific cellular characteristics of the intestine can be diverse among nematode species, they typically conform to polarized epithelial cells with the apical membrane composed of microvilli lining the digestive tube. In apparent contrast to other surfaces of nematodes, digestive and assimilative functions, as well as various metabolic pathways and cellular trafficking, are expected to be extremely active at the intestinal surface. For example, an adult *Caenorhabditis elegans* is capable of producing oocytes with about the same total biomass as its own body per day [1], but the average intestinal residence time for foods was estimated to be less than two minutes in *C. elegans* [2], suggesting that the microvillous membrane must have an enormous capacity for nutrient digestion and absorption. In addition, the intestine has to offer innate immunity against invasive pathogens, and adaptations at the apical intestinal membrane may be required to protect parasitic nematodes against host immune systems. Furthermore, the nematode intestine has been suggested to be involved in other biological processes such as stress responses, body size control, and aging [1].

Three lines of evidence indicate that the intestine is an important target for the control of parasitic nematodes. First, intestinal antigens enriched for apical membrane-associated proteins have been successfully used to immunize against *Haemonchus contortus*, a hematophagous nematode of small ruminants [3]–[7]. Surface-bound nematode proteases are a dominant, but not exclusive, group of proteins that have been implicated in inducing this protection. A prospective mechanism of the immunity involves perturbing nutrient digestion and acquisition at the intestinal surface by the ingested host-derived antibodies capable of neutralizing parasite digestive proteases [7]. Further investigations conducted with hematophagous hookworms also produced similar effects [8]. Second, adult *H. contortus* intestinal cells are hypersensitive to benzimidazole anthelmintics, apparently through the target protein beta-tubulin isotype 1 [9],[10]. It was suggested that the drug inhibited vesicle transport in the apical secretory pathway, causing the intracellular release of the digestive enzymes destined for secretion and subsequent cytotoxic effects [9]. Third, parasite control has been demonstrated by inhibition of an intestinal enzyme, cathepsin L cysteine protease, by either RNA interference or a chemical inhibitor in the plant parasitic nematode *Meloidogyne incognita* [11]. These observations generate great interests to uncover the basic characteristics of the intestinal cells that might be further exploited for the broad control of parasitic nematodes. However, the dearth of relevant experimental systems and molecular information such as gene repertoires for many parasitic species has impeded rapid progress.

Five major clades (I–V) are currently recognized to comprise the phylum Nematoda [12],[13]. So far, almost all studies of the intestine at the gene level have focused on the clade V nematodes. A small-scale sampling of expressed sequence tags (ESTs) from the dissected intestine from adult *H. contortus* females identified 51 intestinal genes including cysteine proteases [14], this list was later expanded via a proteomic approach to include a number of apical intestinal membrane proteases from *H. contortus* and hookworms [15]. Intestinal EST libraries generated from laser-dissected materials from *Necator americanus* and *Ancylostoma caninum* allowed the identification of 544 intestine-expressed genes [16]. Although a more comprehensive dataset with >5,000 intestinal genes is available in *C. elegans* [17]–[19], it is unclear, given the evolutionary diversity within Nematoda, to what extent the molecular and cellular functions of the intestine can be extrapolated across nematode species.

In this study, we sampled the transcribed genomes from several tissues and developmental stages from two parasitic nematodes: the clade III nematode *Ascaris suum*, which presumably feeds on the semi-digested contents in the host intestine, and the clade V blood-feeding parasite *H. contortus*. Nearly 10,000 and 5,000 genes were identified from the two nematodes, respectively. More importantly, given the attention to the intestine, we produced the largest collection of intestinal genes in parasitic nematodes by dissecting adult intestine from each species, a procedure that is not practical for many other nematodes because of their small sizes and the lack of laboratory culturing systems. Extensive cross-species comparisons were made among the adult intestinal genes from the parasites and those expressed in the adult intestine of the free-living bacterivore *C. elegans*. Both diversification and conservation of intestinal gene repertories were evident among the species investigated. The diversities of intestinal transcriptomes by clade and species may reflect the substantial life style differences among these nematodes. A group of 241 protein families were found conserved in the intestine of all three nematodes, accounting for ∼20% of the intestinal gene repertoires from the three species. These genes may include core intestinal functions that are indispensable among many nematodes. Functional annotations were generated for the intestinal genes. Molecular characteristics of the intestinal genes were further explored to highlight various physiological aspects of the nematode intestine.

## Materials and Methods

### EST generation, clustering, and translation

Dissection of the adult intestine was carefully performed under microscopy as described previously [14],[20],[21]. The samples used in this study had also passed another round of visual inspection microscopically to ensure they did not contain other tissues such as muscle, esophagus, or hypodermis. Detailed information on genetic materials and cDNA library construction are available at www.nematode.net. ESTs were processed and clustered as described before [22]–[25]. EST contig sequences were translated individually by Prot4EST, a 6-tier translation pipeline combining both similarity-based methods and *de novo* predictions [26], for downstream analysis.

### Identification of sequence similarities

Databases used for sequence comparisons were*:* i) *Caenorhabditis spp.*, all amino acid sequences in the complete genomes of *C. elegans* (Wormbase Release v150), *C. briggsae* (June, 2006), and *C. remanei* (June, 2006), ii) Other Nematoda, all non-*Caenorhabditis* nematode nucleic acid sequences in GenBank excluding those from *A. suum* (when analyzing *A. suum* sequences) or *H. contortus* (when querying *H. contortus* sequences) (October 18, 2006), and iii) Non-Nematoda, all amino acid sequences in the non-redundant protein database NR excluding those from nematode species (September 20, 2006). WU-BLASTP (wordmask = seg postwe B = 1000 topcomboN = 1) was used to query the translated sequences against protein databases, and WU-TBLASTN (wordmask = seg lcmask B = 1000 topcomboN = 1) for searching against nucleotide databases [27]. The *E-*value cutoff of 1.0e^−5^ was used to accept sequence similarities in all BLAST searches.

### Identification of intestine-enriched genes

Each intestinal EST cluster was assigned two counts according to the numbers of times it was sampled from either the intestinal or non-intestinal cDNA libraries, respectively. Similarly, each *C. elegans* intestinal gene was assigned two counts for the numbers of times it was sampled by SAGE tags from either the *glp-4* dissected gut or the *glp-4* adult whole worm, respectively. The mutants lack the gonad when raised at 25°C, therefore contamination by other tissues is less likely [17]. The SAGE data was downloaded with sequence quality filter = 0.99, no normalization, duplicate ditags and ambiguous or antisense tags removed (April 19, 2006; mapped to Wormbase Release v150) [17]. A Poisson-based enrichment test, considering both the total sampling sizes and random variations [28], was implemented to compute an *P*-value to represent the likelihood of intestinal enrichment for each EST cluster or *C. elegans* gene using these two counts. The *P*-value cutoff of 0.001 was chosen to define the putative intestine-enriched genes from the three nematodes.

### Prediction of signal peptide (SP) and transmembrane (TM) domain

A hidden Markov modeling-based algorithm, Phobius [29], was used with default setting. Each query sequence was further annotated as TM-only, TM with SP, SP-only, or intracellular based on raw Phobius outputs. For each EST cluster, Phobius annotation was predicted for each contig and summarized at the EST cluster level. A modified Wormbase Release v150 containing only the longest splicing isoform at each gene loci was used as the complete gene set of the *C. elegans* genome.

### Identification of orthogous gene pairs between *A. suum* and *C. elegans*

For tissue-level comparisons made between intestine and gonad, InParanoid [30] was used at default settings to identify a total of 1,764 putative orthologous groups between all the *A. suum* EST clusters and the complete gene set of *C. elegans* (the modified Wormbase Release v150 containing only the longest splicing isoform at each gene loci). InParanoid-generated main orthologous pairs, which are essentially the mutual-best matches between all the available genes from the two species, were further screened against the 447,546 *A. suum* Genome Survey Sequences (GSSs) that were generated recently (Mitreva, unpublished), resulting in the final group of 1,652 putative main orthologous pairs in which the *C. elegans* members do not have better matches in GSSs than the *A. suum* EST partners assigned by InParanoid. *C. elegans* gonad-expressed genes were extracted from SAGE data generated from dissected gonad (March 12, 2007) [17].

### Identification of intestinal protein families

An all-against-all WU-BLASTP was performed on all the 9,918 translated intestinal genes from the three species (including sequences for EST contigs from the two parasites and 5,056 *C. elegans* genes). Raw BLAST results were fed to a C-language implementation of Markov Cluster (MCL) Algorithm (www.micans.org/mcl), a fast and scalable unsupervised cluster algorithm based on simulation of flow in graphs [31]. An Inflation Fact of 1.6 was chosen for the MCL clustering. The MCL output was then summarized at the EST cluster level, during which we applied an additional filtering step to remove an EST cluster from a MCL protein family if less than 10% of its total contigs were clustered into that family. These parameters were based on manual inspection of the results on a test set consisting of the putative intestine-enriched genes with 210 parasite EST clusters and 247 *C. elegans* genes (false positive rate of 3%; data not shown).

### Gene Ontology mappings and identification of statistically enriched ontologies

Default parameters for InterProScan v13.1 [32] were used to search against the InterPro database [33]. Raw InterProScan results for the translated EST contigs were summarized at the EST cluster level. Gene ontology (GO) terms were further assigned and displayed graphically by the AmiGO browser with default parameters and the ontology data released on March 15, 2007[34]. Complete GO mappings for the three intestinal transcriptomes are available at www.nematode.net. For each GO term, its enrichment in an IntFam group (such as the IntFam-241 group) was measured over the complete set of 9,918 translated intestinal genes using a hypergeometric test, the *p*-value cutoff of 1.0e^−5^ was chosen for enrichment. The less informative ontologies, including those at level 4 or higher for Biological Process or Molecular Function, and those at level 2 or higher for Cellular Component, were removed from the enrichment list. Also removed were redundant ontologies by keeping only the lower level more informative ontology if the same group of genes was mapped to more than one GO term.

### KEGG pathway analysis

An empirical mixed approach was used for mapping the novel genes to canonical pathways. The *E*-value cut-off of 1.0e^−10^ reported by WU-BLASTP against the Genes Database Release 39.0 from Kyoto Encyclopedia of Genes and Genomes (KEGG) was first used for finding homologous matches. Then the top match and all the matches within a range of 30% of the top BLAST score, if meeting the cut-off, were accepted for valid KEGG associations [35]–[37]. A hypergeometric test, measuring the relative coverage of the KEGG-annotated orthologous groups assigned to a pathway, was implemented to identify the enriched pathways for each intestine [38].

### Accession numbers

Nucleotide sequences data reported in this paper are available in the GenBank, EMBL and DDBJ databases. The accession numbers for ESTs from *A. suum* are: BI781215-BI784439, BM032617-BM034650, BM280443-BM285290, BM318846-BM319958, BM515079-BM518821, BM566483-BM567588, BM568416-BM569529, BM732977-BM734435, BM964439-BM965448, BQ094886-BQ096565, BQ380669-BQ383404, BQ835081-BQ835723, BU965907-BU966430, CA849193-CA850481, CA953713-CA955182, CB100077-CB102042, DV018957-DV019894, EB186562-EB187079. The accession numbers for ESTs from *H. contortus* are: CA033335-CA034379, CA868595-CA870175, CA956361-CA959150, CB018493-CB022024, CB063882-CB065260, CB099467-CB100076, CB190871-CB192419, CB331948-CB333475.

## Results/Discussion

### Generation and Clustering of ESTs from Clade III and V Parasitic Nematodes

We constructed 18 *A. suum* and 6 *H. contortus* stage- or tissue-specific cDNA libraries, and sequenced 31,416 and 14,014 5-prime ESTs from the two species, respectively. These ESTs totaled to 13.6 and 6.3 million bases for *A. suum* and *H. contortus*, accounting for 77% and 63% of the total nucleotides from the two species currently available in public databases (Table S1). Supplemented by 9,354 *A. suum* and 8,146 *H. contortus* ESTs previously deposited in GenBank (retrieved in January, 2006), all available ESTs were grouped into 17,989 *A. suum* and 9,842 *H. contortus* EST contigs, each containing ESTs derived from nearly identical transcripts according to overlapping sequences to reduce sequence redundancy [23],[24]. The contigs were further assembled into 9,947 *A. suum* and 5,058 *H. contortus* EST clusters based on sequence similarities identified among contigs as well as in previously identified genes (Table S1). Each EST cluster likely represents transcripts derived from a single genomic locus and therefore is approximated as one gene [22]–[24]. Given that *C. elegans* and *C. briggsae* each contains ∼19,000 protein-coding loci, and between 14,500 and 17,800 genes were inferred from the *Brugia malayi* draft genome [39], we have consequently identified a substantial portion of the complete gene sets from the two parasites. These data will vastly facilitate the genome assembly and annotation in the related nematode genome sequencing projects currently underway. Initial investigation of the identities of these novel genes was performed by comparing the translated sequences with known proteins from other organisms (Text S1; Figure S1).

### Intestinal Transcriptomes from Adult *A. suum*, *H. contortus*, and *C. elegans*

To study the intestinal transcriptomes, four cDNA libraries (out of the 18) from *A. suum* and three (out of the 6) from *H. contortus* were constructed from dissected adult intestine with methods based on either Poly-A [40] or spliced leader sequences [24]. Among all the ESTs we generated, a total of 9,586 *A. suum* and 7,068 *H. contortus* ESTs were derived from these intestinal libraries. These ESTs occurred in 3,121 *A. suum* and 1,755 *H. contortus* EST clusters, accounting for about 30% of the total genes sampled in each nematode. Since these EST clusters contained ESTs sampled from the adult intestine, they were considered to represent adult intestinal genes, making this the largest tissue-level gene discovery in parasitic nematodes thus far (Table 1).

**Table 1:** Intestinal Transcriptomes and Intestine-Enriched Genes from Three Nematodes.

Summary	*A. suum*	*H. contortus*	*C. elegans*
**cDNA libraries**			
Non-intestinal	14	3	
Intestinal	4	3	
**ESTs/SAGE Tags ** 1			
Total	38,978	22,152	104,756
Non-intestinal	29,392	15,084	53,197
Intestinal	9,586	7,068	51,559
**EST Clusters/Genes**			
Total	9,947	5,058	
Non-intestinal	6,826	3,303	
Intestinal	3,121	1,755	5,065
Int. genes w/Gene Ontology	954	620	2,911
% Int. genes w/Gene Ontology	31%	35%	57%
**Intestine-enriched EST Clusters/Genes ** 2			
* p*-value < = 0.001	150 (10)	60 (5)	247 (7)

1 SAGE tags from *glp-4* adults (6,903 genes) and dissected gut (4,071 genes) for *C. elegans.*

2 Included in parentheses are putative false positives.

In contrast to the two gastrointestinal parasites, the free-living model nematode *C. elegans* is a bacterivore obtaining nutrients primarily or exclusively from the consumption of bacteria. Two previous studies reported identification of genes expressed in the adult *C. elegans* intestine: i) sequence tags generated by serial analysis of gene expression (SAGE) from the dissected adult intestine were mapped to over 4,000 *C. elegans* genes [17],[18]; ii) a study using mRNA tagging and microarray gene expression profiling identified ∼1,900 intestine-expressed genes [19]. Consolidating the two efforts provided us with a non-redundant set of 5,065 intestinal genes from adult *C. elegans*, covering over 25% of all coding loci in its entire genome (Table 1).

The phylum Nematoda is ancient and diverse. Even though the evolutionary distance between clade III *A. suum* and clade V *C. elegans* was estimated to be ∼350 million years [41], the nematode intestine has maintained high similarity in both tissue morphology and presumably physiology (i.e. involvement in feeding). However, it is unknown how much the intestine is conserved, or diversified, at the molecular level across species. The tissue-level gene sampling in this study offered an opportunity to investigate this question.

### Diversification among Intestinal Transcriptomes

**Figure 1:**
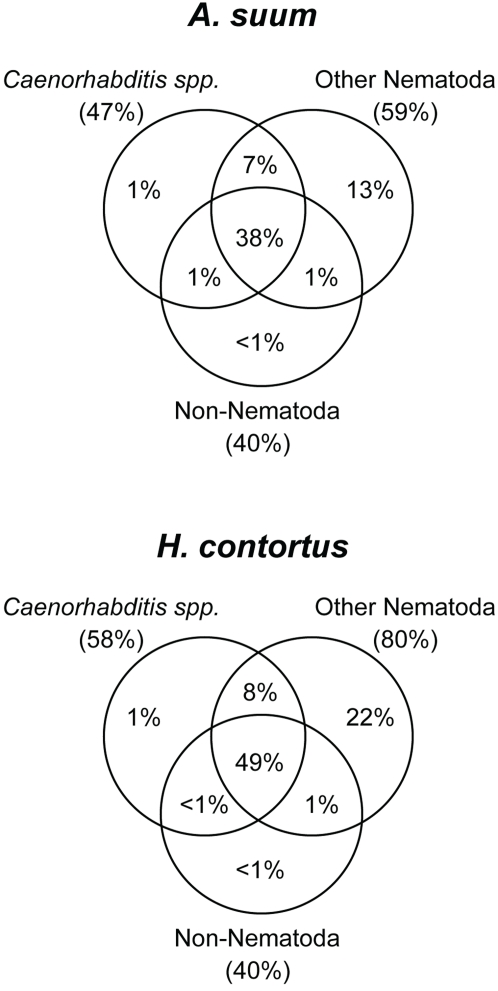
Sequence Similarities Identified in the *A. suum* and *H. contortus* Intestinal Transcriptomes. The three phylogenetically specific sequence groups used to identify sequence similarities of the intestinal genes were: i) *Caenorhabditis spp.*, amino acid sequences from the complete genomes of *C. elegans*, *C. briggsae*, and *C. remanei*, ii) Other Nematoda, non-*Caenorhabditis* nematode nucleic acid sequences excluding those from either *A. suum* or *H. contortus*, when sequences from *A. suum* or *H. contortus* were queried, respectively, and iii) Non-Nematoda, non-nematode amino acid sequences from the non-redundant protein database NR. In total, 61% (1,893/3,121) *A. suu*m and 81% (1,423/1,755) *H. contortus* intestinal genes contained primary sequence similarities to known proteins from other organisms.

Differences in the intestinal gene repertoires were obvious among the three nematodes. In total, 39% of *A. suum* and 19% of *H. contortus* intestinal genes were found to be novel compared to all known proteins in the public databases (Figure 1). Such novel intestine-expressed parasite genes contained no match in the complete genome of the free-living *C. elegans*, thus not in the *C. elegans* intestine, making them unique by comparison to *C. elegans*. In addition, for the sampled intestinal genes from both parasites, the non-*Caenorhabditis* nematodes offered the largest numbers of homologous matches than either the *Caenorhabditis* species or the non-nematode organisms (Figure 1). Such differences may suggest the existence of lineage- or species-specific diversification in the nematode intestine.

**Figure 2:**
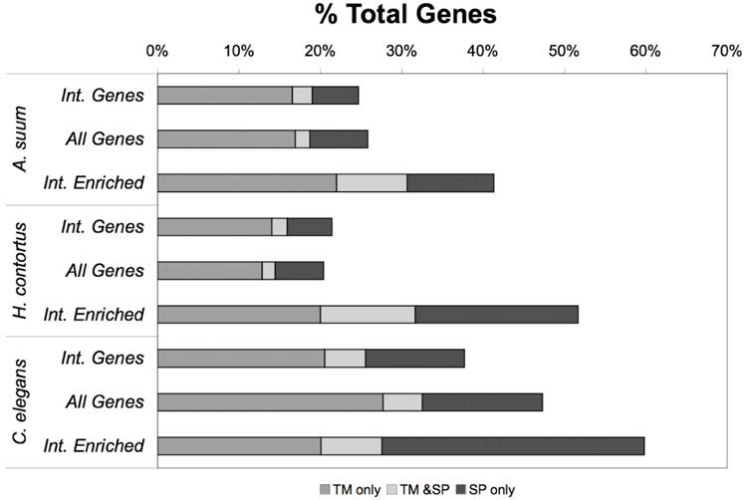
Putative Secreted or Trans-membrane Proteins in *A. suum*, *H. contortus*, and *C. elegans*. Larger percentages of the putative intestine-enriched genes (Int. Enriched) were predicted to be secreted with signal peptide (SP) or trans-membrane (TM) than either the complete set of intestinal genes (Int. Genes) or the complete set of all available genes (All Genes) in *A. suum*, *H. contortus*, and *C. elegans*.

Furthermore, we observed higher levels of diversification in the putative intestine-enriched genes from the three nematodes. Taking into consideration sample size and random sampling fluctuation [28], we identified 150 *A. suum*, 60 *H. contortus*, and 247 *C. elegans* putative intestine-enriched genes based on the “digital” expression levels revealed in EST and SAGE data (at the Poisson distribution-based *P*-value cutoff of 0.001) (Table S2; Table S3; Table S4). Many of these predicted enrichments suggested unique intestinal functions for the individual species. For example, the group of 60 genes from the blood-feeding *H. contortus* includes 2 fibrinogen-related proteins that may function as thrombin inhibitors to prevent clotting of ingested blood. Also included are putative enzymes that may be involved in the digestion of hemoglobin, one of the major food sources of blood-feeding parasites, including a serine-type protease, a metallopeptidase, and 13 different cysteine-type proteases that were reported previously [42] (Table S3). Interestingly, a significantly higher percentages of these genes (e.g. 15%-31% higher than all the sampled intestinal genes) encode proteins predicted as secreted or trans-membrane [29] (Figure 2), suggesting that they interact with the extracellular environment. However, 64%, 54%, and 69% of them, from the three species respectively, were distinct from members of the protein families conserved in the intestine of all three nematodes (IntFam-241; see below), indicating that a large portion of these putative intestine-enriched genes are specific to the intestine of individual nematode lineages or species. This further underlines the diversification of intestinal transcriptomes in accommodating the different life styles and feeding patterns among nematodes.

### Molecular Conservation of the Nematode Intestine

To evaluate common characteristics of the nematode intestine, we first sought evidence for the molecular conservation of the tissue in the context of phylogeny. We made comparisons among genes expressed in the intestine of *A. suum* and *C. elegans* and those expressed in another tissue, namely the gonad. These two species have the largest numbers of sequences available, and they also represent the most distant relationship among the three nematodes investigated. The gonad was chosen because the next largest group of genes was sampled from this tissue in *A. suum* after the intestine. *H. contortus* was excluded from this analysis because a gonad-expressed gene set was not available from this nematode. Genes expressed in the intestine and gonad were divided into four putative tissue-specific groups: i) 2,453 *A. suum* and ii) 2,557 *C. elegans* genes expressed in the intestine but not in the gonad (the two intestine groups), and iii) 2,690 *A. suum* and iv) 2,589 *C. elegans* genes that were found in the gonad but not in the intestine (the two gonad groups). The use of the similar numbers of genes in each group is expected to reduce false results caused by over-representation from any single category.

Molecular conservation was first evaluated by comparing the numbers of putative homologous pairs identified among the intestine and gonad gene groups. The number of the putative homologs between the two intestine groups was significantly larger than that between the intestine and gonad groups (*p*-value = 2.5e^−04^ at the bit-score cutoff of 100 in a permutation two-tailed Z-test; *p*-value = 4.2e^−08^ at the bit-score cutoff of 50; Figure S2), suggesting that for genes expressed in the intestine of one nematode, their homologous matches in another species are significantly more likely to be expressed in the intestine than in the gonad of the second nematode. These results provide evidence for the molecular conservation of the intestine across these distantly related nematodes. In contrast, the number of putative homologs between the two gonad groups was not statistically different from that between the gonad and intestine (Figure S2), indicating that the gonad genes appeared to be less conserved than those expressed in the intestine in this two-tissue comparison.

**Figure 3:**
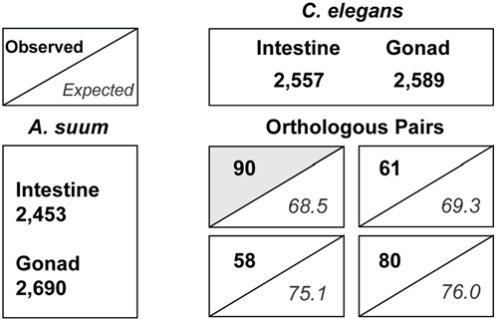
Orthologous Genes Tend to Maintain Their Intestinal Expression Patterns across *A. suum* and *C. elegans*. A total of 289 putative orthologous pairs were identified among the intestine or gonad gene groups from *A. suum* and *C. elegans*. Ninety such pairs were found between the two intestine gene groups, representing an enrichment of 31% over the expectation from a random distribution of orthologous pairs, and an enrichment of only 5% was detected between genes in the two gonad groups. The null hypothesis of random orthologous pairing was rejected at a confidence level of at least 99% with a χ^2^ value of 11.9 between the observations and expectations.

To increase the confidence of analysis, we next focused on the putative orthologous pairs predicted among the intestine and gonad gene groups, which was a smaller data set than the homologous pairs used above but with higher stringency. Among the total of 1,652 putative orthologous pairs predicted from *A. suum* and *C. elegans* (see Materials and Methods), 289 were paired among genes from the intestine and gonad groups. They were used in a Chi Square statistical test, with random distribution of orthologous pairs as the null hypothesis. Compared to the expected numbers, there was a 31% enrichment of orthologous pairs observed between the *A. suum* and *C. elegans* intestine groups (Figure 3), whereas the enrichment between the two gonad groups was only marginal (5%), and the observed numbers of orthologous pairs between the gonad and intestine groups were less than expected (Figure 3). Overall, a significant χ^2^ value of 11.9 rejects the null hypothesis at a confidence level higher than 99% (*p*-value <0.01) [43], and selective pressure is evident on molecular conservation of the intestinal gene repertories.

Although the use of the incomplete transcriptomes and a bias towards relatively abundant transcripts in EST sampling can affect results, analyses of either homologous or orthologous pairs both provide direct support for the molecular conservation of the nematode intestine. With the obvious pattern of diversification in the nematode intestine (discussed earlier), our results indicate that a subset of the intestinal gene repertoires, which likely contribute to the intestinal characteristics conserved across diverse nematode species, remain conserved during the evolution of Nematoda.

Interestingly, genes expressed in the gonad appear to be less well conserved based on both analyses. However, these results do not suggest the lack of evidence for the conservation of the gonad. Instead, the two-tissue comparisons indicate that the levels of conservation are lower in the gonad than in the intestine, suggesting that the levels of molecular conservation may differ in different nematode tissues. In fact, the conserved characteristics of the gonad may become more evident with larger sample sizes and/or by comparisons with another tissue with a lower level of conservation than the intestine, when new sequence data becomes available. Similarly, differences at the levels of molecular conservation were observed in different tissues between human and mouse, which diverged only about 25 million years ago [44]. Future comparisons with more complete expression data across multiple tissues in different nematode species should offer additional insights into this aspect of nematode evolution.

### Identification of “Core” and Other Groups of Intestinal Protein Families

**Figure 4:**
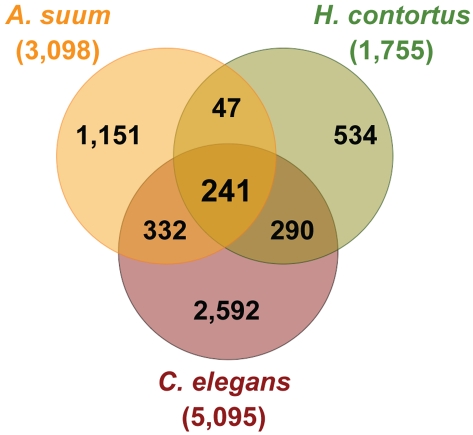
Protein Families in the Adult Intestine from *A. suum*, *H. contortus*, and *C. elegans*. In total, 5,587 intestinal protein families (IntFam) were built from the complete set of 9,918 translated intestinal genes sampled from the three species. Forty-one percent of all the intestinal genes were grouped into 910 multiple-species IntFam groups; 2,024 genes, including 752 from *A. suum*, 455 from *H. contortus*, and 817 from *C. elegans*, were found in a group of 241 families (IntFam-241) containing members from all three intestines. The IntFam-241 families likely represent an ancestral intestinal transcriptome involved in core cellular and physiological intestinal functions common to the investigated species or even to Nematoda, they are thus referred as the “core” IntFam-241 group.

To compare the intestinal transcriptomes of *A. suum*, *H. contortus*, and *C. elegans* in a single analysis, we built protein families from the complete set of 9,918 translated intestinal genes combined from the three nematodes. A total of 5,587 intestinal protein families (IntFam) were identified conservatively based on sequence similarities by MCL clustering [31] (Figure 4). Proteins assigned into the same protein family contain putative homologous or orthologous matches among the three species. Both diversification and conservation of the intestinal transcriptomes was obvious at the protein family level in this 3-species comparison. A total of 59% of all the sampled intestinal genes were members of the protein families containing proteins from only one nematode (Figure 4). Although the assignments for many of these single-species families are likely to change when more complete intestinal gene repertories become available, this group includes the genes contributing to the unique intestinal features in each species. The remaining 41% of the intestinal genes formed 910 multi-species protein families; they are conserved in the intestine of at least two nematodes. Among these multi-species families, 241 had members from all three species, accounting for ∼20% of all the intestinal genes under investigation (Figure 4). Given the differences in life styles and feeding patterns among the three nematodes, we propose that these 241 intestinal protein families represent an ancestral intestinal transcriptome involved in core cellular and physiological intestinal functions common to the investigated species or even across the Nematoda. Therefore, we referred to them as the “core” IntFam-241 group.

### Functional Analysis of Intestinal Genes Based on Gene Ontology

The 9,918 translated intestinal genes sampled from the three nematodes were annotated and classified using Gene Ontology [34],[45]. Ontologies were assigned at a higher ratio (58%) to the *C. elegans* intestinal genes than to those from *A. suum* (31%) or *H. contortus* (35%; Table 1). In addition, genes in the multi-species IntFam groups, which contained members from at least two nematodes, were annotated at higher ratios (47%–74%), whereas only 8% of the genes were annotated from the two single-species IntFam groups containing members only from *A. suum* or *H. contortus* (data not shown). These data may indicate that novel intestinal genes have independently evolved in relation to the different lineages of parasitism. Complete GO mappings for the three intestinal transcriptomes are presented in the searchable AmiGO browser at www.nematode.net [46]. Furthermore, A hypergeometric test was implemented to identify ontologies that are statistically enriched, thus indicating enriched features, in the core IntFam-241 (Table 2) as well as other IntFam groups (Text S1; Table S6).

**Table 2:** Gene Ontology Terms Statistically Enriched among 2,024 Intestinal Genes in IntFam-241.

GO id	IntFam-241 Genes Mapped 1				All Int. Genes Mapped	*P*_value	Gene Ontology
	*As*	*Hc*	*Ce*	Total			
**GO:0008150**							**biological_process**
GO:0044267	222	165	246	633	1,081	0.0E+00	cellular protein metabolic process
GO:0006412	154	62	74	290	451	0.0E+00	translation
GO:0006464	36	24	104	164	297	0.0E+00	protein modification process
GO:0006796	34	24	103	161	284	0.0E+00	phosphate metabolic process
GO:0016310	29	23	99	151	240	0.0E+00	phosphorylation
GO:0043687	28	18	101	147	243	0.0E+00	post-translational protein modification
GO:0006468	16	9	82	107	149	0.0E+00	protein amino acid phosphorylation
GO:0007264	8	10	28	46	63	9.92E-11	small GTPase mediated signal transduction
GO:0006457	12	9	22	43	62	5.95E-09	protein folding
GO:0006508	19	69	49	137	281	2.50E-08	proteolysis
GO:0042254	5	7	9	21	25	2.46E-07	ribosome biogenesis and assembly
GO:0006414	9	5	9	23	29	4.69E-07	translational elongation
GO:0006512	7	9	13	29	43	4.62E-06	ubiquitin cycle
GO:0046034	11	11	16	38	62	5.33E-06	ATP metabolic process
**GO:0005575**							**cellular_component**
GO:0005737	172	72	123	367	697	0.0E+00	cytoplasm
GO:0044444	164	68	95	327	590	0.0E+00	cytoplasmic part
GO:0005840	145	52	58	255	371	1.52E-12	ribosome
GO:0030529	149	58	67	274	408	1.81E-12	ribonucleoprotein complex
GO:0043232	161	58	69	288	480	2.36E-12	intracellular non-membrane-bound organelle
GO:0015935	8	5	8	21	24	5.74E-08	small ribosomal subunit
GO:0005740	10	9	19	38	61	3.03E-06	mitochondrial envelope
GO:0005874	4	3	4	11	11	5.55E-06	microtubule
GO:0044429	10	9	21	40	67	7.61E-06	mitochondrial part
GO:0005739	13	12	26	51	92	9.02E-06	mitochondrion
**GO:0003674**							**molecular_function**
GO:0030554	44	24	161	229	468	0.0E+00	adenyl nucleotide binding
GO:0016773	18	10	84	112	186	0.0E+00	phosphotransferase activity, alcohol group as acceptor
GO:0004672	16	9	82	107	151	0.0E+00	protein kinase activity
GO:0004674	13	8	63	84	103	0.0E+00	protein serine/threonine kinase activity
GO:0005525	15	17	40	72	103	0.0E+00	GTP binding
GO:0004713	7	6	26	39	46	0.0E+00	protein-tyrosine kinase activity
GO:0000062	29	1	6	36	36	0.0E+00	acyl-CoA binding
GO:0004194	5	14	12	31	32	0.0E+00	pepsin A activity
GO:0005524	44	25	156	225	436	1.98E-13	ATP binding
GO:0016301	20	11	88	119	213	1.04E-12	kinase activity
GO:0004190	6	14	12	32	35	3.79E-12	aspartic-type endopeptidase activity
GO:0004197	5	44	16	65	95	4.26E-12	cysteine-type endopeptidase activity
GO:0008234	5	46	18	69	105	1.02E-11	cysteine-type peptidase activity
GO:0004175	15	59	33	107	190	2.75E-11	endopeptidase activity
GO:0015035	8	6	15	29	41	9.97E-07	protein disulfide oxidoreductase activity
GO:0005544	9	1	2	12	12	1.84E-06	calcium-dependent phospholipid binding
GO:0016620	6	2	6	14	15	2.10E-06	oxidoreductase activity, acting on the aldehyde or oxo group of donors, NAD or NADP as acceptor

1 *As*: *A. suum*; *Hc*: *H. contortus*; *Ce*: *C. elegans*; Total: IntFam-241 genes from all the three species.

Five of the 17 enriched Molecular Function ontologies in IntFam-241 are related to protein kinases (Table 2; Table 3). Protein kinases are one of the largest and most influential protein families, accounting for about 2% of genes in a variety of eukaryotic genomes including *C. elegans* and *B. malayi*. They regulate almost every aspect of cellular activities and may phosphorylate up to 30% of entire proteomes [39],[47]. Based on GO annotations, protein kinases were enriched by ∼3.5 fold in IntFam-241 over the complete set of intestinal genes (5.3% vs. 1.5% of the total genes for each group). Both serine/threonine- and tyrosine-types of protein kinases were found to be enriched. Novel protein kinases from the parasites were further classified based on their *C. elegans* homologs (Table S5). Interestingly, molecular functions such as adenyl nucleotide binding, ATP binding, and GTP binding were also enriched. The involvement of these functions in protein kinase activities further suggested key roles of cellular signaling in the nematode intestine (Table 2).

**Table 3:** Selected Gene Ontology Annotations in IntFam-241.

GO Annotation	GO id	Genes
**Protein Kinases**		
protein kinase activity	GO:0004672	107
**Serine/threonine-specific protein kinases**		
protein serine/threonine kinase activity	GO:0004674	84
receptor signaling protein serine/threonine kinase activity	GO:0004702	4
MAP kinase activity	GO:0004707	4
**Tyrosine-specific protein kinases**		
protein-tyrosine kinase activity	GO:0004713	39
transmembrane receptor protein tyrosine kinase activity	GO:0004714	2
epidermal growth factor receptor activity	GO:0005006	1
vascular endothelial growth factor receptor activity	GO:0005021	1
**Protein Phosphatases**		
phosphoprotein phosphatase activity	GO:0004721	10
protein tyrosine phosphatase activity	GO:0004725	10
**Proteases**		
peptidase activity	GO:0008233	137
**Serine proteases**		
serine-type peptidase activity	GO:0008236	9
**Threonine proteases**		
threonine endopeptidase activity	GO:0004298	8
**Cystine proteases**		
cysteine-type peptidase activity	GO:0008234	69
cysteine-type endopeptidase activity	GO:0004197	65
legumain activity	GO:0001509	3
**Aspartic acid proteases**		
aspartic-type endopeptidase activity	GO:0004190	32
pepsin A activity	GO:0004194	31
**Metalloproteases**		
metallopeptidase activity	GO:0008237	16
metalloendopeptidase activity	GO:0004222	1
metalloexopeptidase activity	GO:0008235	11
leucyl aminopeptidase activity	GO:0004178	1
membrane alanyl aminopeptidase activity	GO:0004179	7
metallocarboxypeptidase activity	GO:0004181	3
**Protease Inhibitors**		
protease inhibitor activity	GO:0030414	17
serine-type endopeptidase inhibitor activity	GO:0004867	10
cysteine protease inhibitor activity	GO:0004869	7

The other major Molecular Function terms enriched in IntFam-241 were the proteases (Table 2; Table 3). All but one of the six subtypes of proteases (glutamic acid-type proteases as the exception) had been identified in IntFam-241 (Table 3), suggesting conservation of essential protease functions, such as nutrient digestion and acquisition, among the three species or even across many species of Nematoda. Because each species feeds on distinct food sources, it is possible that related digestive proteases have evolved within each species to adapt for digestion of the different food types. Given the success of parasite control achieved by immunization with *H. contortus* and hookworm intestinal protease-type antigens [3],[8], these proteases may warrant further investigation in *A. suum* and other parasites.

Analysis of the IntFam groups other than IntFam-241 was also conducted. However, in absence of deeper sampling of the intestinal transcriptomes, it is difficult to interpret the results in relation to broadly conserved or lineage- and species-specific characteristics (Text S1; Table S6).

### KEGG-based Pathway Analysis of Three Intestinal Transcriptomes

To identify the biological pathways that are active in the nematode intestine, we mapped the 9,918 intestinal translated sequences, and for comparison, the complete *C. elegans* genes (Wormbase Release v150), to the reference canonical pathways in Kyoto Encyclopedia of Genes and Genomes [35]–[37] (Table 4). Complete listing of all KEGG mappings including graphical representation is available for navigation at www.nematode.net [46].

**Table 4:** KEGG Pathway Mappings for Intestinal Genes from Three Nematodes.

KEGG Pathway	*A. suum*			*H. contortus*			*C. elegans*			Wormbase v150	
	Genes	KOs 1	*P-*value 2	Genes	KOs	*P-*value	Genes	KOs	*P-*value	Genes	KOs
**1. Metabolism**	247	176		167	152		414	316		788	480
1.1 Carbohydrate Metabolism	79	57	6.9E-03	46	45	4.3E-02	123	88	4.7E-03	230	122
1.2 Energy Metabolism	105	70	4.8E-05	80	70	6.7E-09	117	96	6.4E-03	182	135
1.3 Lipid Metabolism	25	22		22	20		71	41	2.3E-02	133	55
1.4 Nucleotide Metabolism	25	20		9	8		37	36		92	57
1.5 Amino Acid Metabolism	42	35		35	29		106	84		209	129
1.6 Metabolism of Other Amino Acids	23	13		11	7		51	30	2.6E-02	91	39
1.7 Glycan Biosynthesis and Metabolism	8	8		6	5		30	21		72	50
1.8 Biosynthesis of Polyketides and Nonribosomal Peptides	0	0		0	0		2	2		3	2
1.9 Metabolism of Cofactors and Vitamins	19	14		17	16		47	31		98	47
1.10 Biosynthesis of Secondary Metabolites	11	9		5	4		24	14		58	21
1.11 Xenobiotics Biodegradation and Metabolism	21	13		14	10		58	26	6.1E-04	120	29
1.12 Enzyme Families	0	0		0	0		0	0		0	0
**2. Genetic Information Processing**	280	133		135	120		248	215		432	336
2.1 Transcription	11	10		18	16		22	21		55	50
2.2 Translation	218	91	5.3E-10	79	73	6.0E-07	130	115	1.6E-04	194	155
2.3 Folding, Sorting and Degradation	50	31		35	28		88	71	6.9E-02	149	104
2.4 Replication and Repair	1	1		3	3		8	8		34	27
**3. Environmental Information Processing**	76	55		42	36		170	99		940	207
3.1 Membrane Transport	11	5		4	4		16	9		47	18
3.2 Signal Transduction	44	27		23	22		79	61		168	103
3.3 Signaling Molecules and Interaction	32	28		19	14		86	37		752	107
**4. Cellular Processes**	75	53		86	39		105	85		254	153
4.1 Cell Motility	16	11		5	3		17	14		39	28
4.2 Cell Growth and Death	9	8		6	6		24	22		51	42
4.3 Cell Communication	24	15		16	11		37	31		114	57
4.4 Endocrine System	18	15		13	13		33	27		68	42
4.5 Immune System	32	18	3.1E-02	58	14	9.9E-02	21	16		55	34
4.6 Nervous System	6	4		6	6		12	11		56	18
4.7 Sensory System	1	1		1	1		4	4		9	5
4.8 Development	5	5		2	2		10	10		22	20
4.9 Behavior	1	1		0	0		1	1		3	2
**5. Human Diseases**	9	8		11	9		17	15		42	30
5.1 Neurodegenerative Disorders	6	5		9	8		12	11		32	21
5.2 Metabolic Disorders	3	3		2	1		6	5		12	11
**7. Unclassified**	44	26		19	15		72	49		221	74
7.1 Metabolism	0	17		13	10		49	35	3.6E-02	145	47
7.2 Genetic Information Processing	18	0		0	0		0	0		1	1
7.3 Cellular Processes and Signaling	0	9		6	5		23	14		75	26
7.4 Poorly Characterized	0	0		0	0		0	0		0	0
**Total Mapped Genes**	660	406		419	334		905	687		2393	1125

1 Each KEGG Orthology (KO) represent an ortholog/paralog group defined by KEGG.

2 Only *P*-values meeting the cutoff of 0.1 are shown here.

The enrichment of specific major KEGG pathways was evident for each intestine by comparisons to the complete KEGG mappings for all *C. elegans* genes (Table 4) [38]. Carbohydrate metabolism, energy metabolism, and translation were identified as the statistically enriched pathways in all three intestinal transcriptomes (at the *p*-value cutoff of 0.05). Interestingly, immune system was an enriched KEGG cellular process in the *A. suum* intestine; this pathway barely missed the cutoff for enrichment in *H. contortus* (with a *p*-value of 9.9e^−02^), but no enrichment was indicated for the *C. elegance* intestine (Table 4). The KEGG immune system was built based on studies in mammalian systems. Many of those from the two parasites were mapped to intracellular proteins of immune cells involved in, for example, intracellular signaling or antigen processing (Table S7; Table S8). Therefore, the potential for their involvement in interactions with the host are not a primary suggestion here, but it cannot be completely excluded either.

### *C. elegans* RNA Interference and Intestinal Genes

RNA interference (RNAi) has been developed and successfully applied to genome-wide gene silencing to inhibit gene functions in *C. elegans* [48]–[51]. *C. elegans* RNAi information can be further extrapolated in understanding functions of orthologous genes in other nematodes, especially in parasitic nematodes where high-throughput screening is not yet practical [52]. For the 3,455 IntFam protein families containing *C. elegans* genes, observed RNAi phenotypes for their *C. elegans* members (Wormbase Release v150) were extracted and extrapolated to a total of 45% of these IntFams (Table 5). Protein families from the IntFam-241 were assigned at a higher ratio (73%) than those from other IntFam groups with *C. elegans* members (Table 5). Among the IntFams-241 families with RNAi phenotypes assigned, 74% (131/176) had severe phenotypes including embryonic, larval, or adult lethal, sterile, sterile progeny, and larval or adult growth arrest (data not shown). Since the IntFam-241 families represent proteins conserved in all the three species, these results further support our hypothesis that the core IntFam-241 protein families likely play key roles in the nematode intestine across many species.

**Table 5:** RNA interference (RNAi) Phenotypes Assigned to IntFams through *C. elegans* Members.

IntFam Group	Families	Families w/RNAi 1	Families w/Pheno 2	% Families w/Pheno
IntFam-241	241	241	176	73%
IntFam_*As*_*Ce*	332	323	182	55%
IntFam_*Hc_Ce*	290	286	168	58%
IntFam_*Ce*_only	2,592	2,488	1,037	40%
All *Ce*-containing IntFams	3,455	3,338	1,563	45%

1 Families whose *C. elegans* members have RNAi results available.

2 Families whose *C. elegans* members have RNAi phenotypes reported.

### Summary

We have performed large-scale sampling of the transcribed genomes in *A. suum* and *H. contortus* from various tissues or developmental stages, accounting for 77% and 63% of total available bases for the two nematodes, respectively. The identification of 9,947 *A. suum* and 5,058 *H. contortus* genes in this study will vastly facilitate the related genome sequencing projects currently underway. The research has produced the largest samplings of the adult intestinal transcriptomes thus far in parasitic nematodes by identifying 3,121 *A. suum* and 1,755 *H. contortus* intestinal genes, making possible the extensive comparative studies with the adult intestinal transcriptome of the free-living *C. elegans*. We found that, even with the evolutionary distance of an estimated 350 million years separating clades III and V nematodes [41], both significant conservation and diversification of gene repertories were evident for the intestine. A group of 241 intestinal protein families, each containing members from all three intestines, were further identified. The IntFam-241 group, containing ∼20% of all intestinal genes sampled from the three species, was proposed to represent an ancient intestinal transcriptome responsible for core cellular and physiological intestinal functions that are conserved in the investigated species or many other nematodes. In addition, various aspects of nematode intestinal physiology were revealed by GO and KEGG classifications of the intestinal transcriptomes, and the examination and extrapolation of available RNAi phenotypes from *C. elegans*. Overall, this study has contributed to a better understanding of nematode biology, providing central information for the development of novel and more effective parasite control strategies. Finally, the use of the *C. elegans* model to dissect basic parasite biology has been slow to evolve. Results presented here identified numerous specific areas of research where *C. elegans* might contribute in this way.

## Supporting Information

Figure S1 Distribution of Sequence Similarities Identified in *A. suum* and *H. contortus* EST Clusters. The three phylogenetically specific sequence groups used to identify sequence similarities of the intestinal genes were: i) *Caenorhabditis* spp., amino acid sequences from the complete genomes of *C. elegans, C. briggsae*, and *C. remanei*, ii) Other Nematoda, non-Caenorhabditis nematode nucleic acid sequences excluding those from either *A. suum* or *H. contortus*, when sequences from *A. suum* or *H. contortus* were queried, respectively, and iii) Non-Nematoda, non-nematode amino acid sequences from the non-redundant protein database NR. In total, 53% (5,303/9,947) *A. suum* and 75% (3,792/5,058) *H. contortus* EST clusters contained primary sequence similarities to known genes from other species, but similar distributions of the identified matches to various species groups were observed in the two parasites.
(0.05 MB PPT)

Figure S2 Homologous Pairs between the Intestine and Gonad Gene Groups from *A. suum* and *C. elegans*. Significant larger number of genes in the *A. suum* intestine group had homologous counterparts in the *C. elegans* intestine group than in the *C. elegans* gonad group at BLAST bit-score cutoff of either 50 or 100, indicating the intestinal expression of homologous genes tend to be maintained across nematodes. However, the number of homologous pairs detected between the two gonad groups was not different from that between the gonad and intestine groups.
(0.14 MB PPT)

Table S1 EST Generation and Clustering.
(0.02 MB XLS)

Table S2 Identification of 150 Intestine-enriched Genes in *A. suum*.
(0.06 MB XLS)

Table S3 Identification of 60 Intestine-enriched Genes in *H. contortus.*
(0.04 MB XLS)

Table S4 Identification of 247 Intestine-enriched Genes in *C. elegans.*.
(0.07 MB XLS)

Table S5 Classification of Putative Protein Kinases in *A. suum* and *H. contortus.*
(0.03 MB XLS)

Table S6 Gene Ontology Terms Statistically Enriched in IntFam Groups Other Than IntFam-241.
(0.03 MB XLS)

Table S7 Genes Mapped to KEGG Immune System in *A. suum.*
(0.03 MB XLS)

Table S8 Genes Mapped to KEGG Immune System in *H. contortus.*
(0.03 MB XLS)

Text S1 Supplementary Materials.
(0.04 MB DOC)
