# Supplementary material for: Intestinal Transcriptomes of Nematodes: Comparison of the Parasites Ascaris suum and Haemonchus contortus with the Free-living Caenorhabditis elegans
Source: PLoS Negl Trop Dis. 2008 Aug 6;2(8):e269. doi: 10.1371/journal.pntd.0000269 (PMC2483350; doi:10.1371/journal.pntd.0000269)
Supplement: Figure S1 — Distribution of Sequence Similarities Identified in A. suum and H. contortus EST Clusters. The three phylogenetically specific sequence groups used to identify sequence similarities of the intestinal genes were: i) Caenorhabditis spp., amino acid sequences from the complete genomes of C. elegans, C. briggsae, and C. remanei, ii) Other Nematoda, non-Caenorhabditis nematode nucleic acid sequences excluding those from either A. suum or H. contortus, when sequences from A. suum or H. contortus were queried, respectively, and iii) Non-Nematoda, non-nematode amino acid sequences from the non-redundant protein database NR. In total, 53% (5,303/9,947) A. suum and 75% (3,792/5,058) H. contortus EST clusters contained primary sequence similarities to known genes from other species, but similar distributions of the identified matches to various species groups were observed in the two parasites. (0.05 MB PPT) [file pntd.0000269.s001.ppt]

## Slide 1
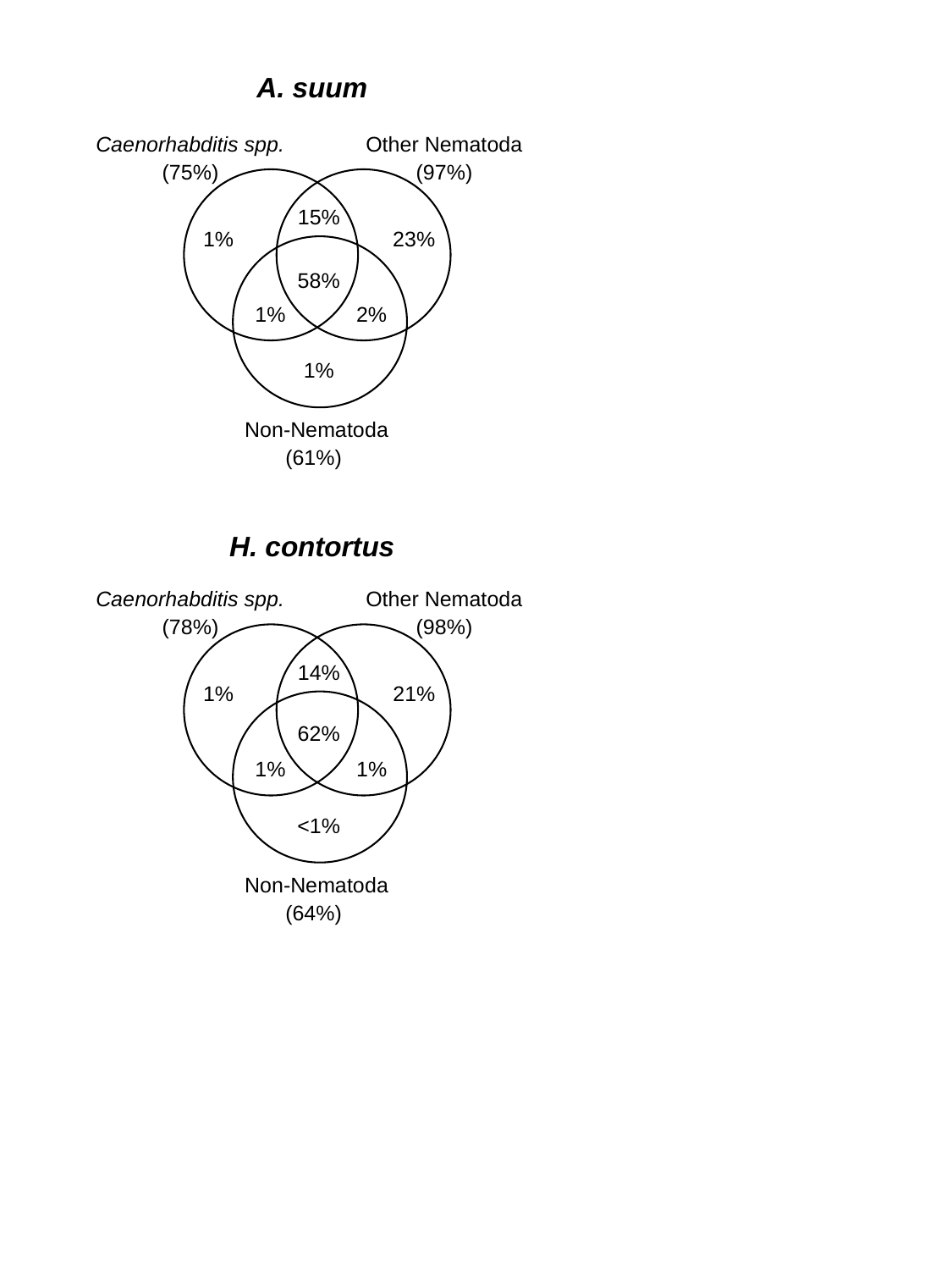

A. suum
Caenorhabditis spp. (75%)
Other Nematoda (97%)
15%
1%
23%
58%
2%
1%
1%
Non-Nematoda
(61%)
H. contortus
Caenorhabditis spp. (78%)
Other Nematoda (98%)
14%
1%
21%
62%
1%
1%
<1%
Non-Nematoda
(64%)
