# Supplementary figures and images for: Intestinal Transcriptomes of Nematodes: Comparison of the Parasites Ascaris suum and Haemonchus contortus with the Free-living Caenorhabditis elegans
Source: PLoS Negl Trop Dis. 2008 Aug 6;2(8):e269. doi: 10.1371/journal.pntd.0000269 (PMC2483350; doi:10.1371/journal.pntd.0000269)

## Slide 1
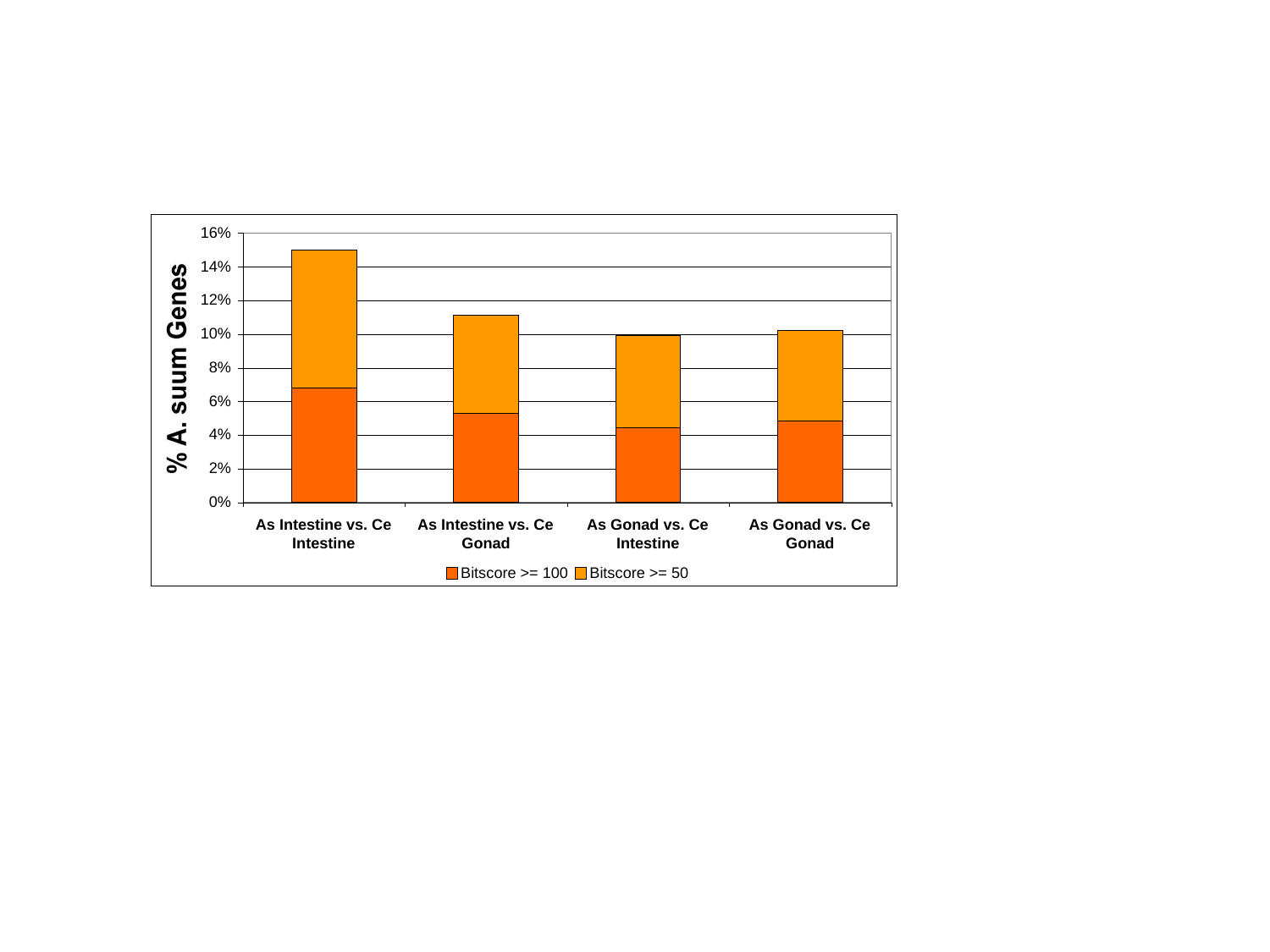

Supplement: Figure S2 — Homologous Pairs between the Intestine and Gonad Gene Groups from A. suum and C. elegans. Significant larger number of genes in the A. suum intestine group had homologous counterparts in the C. elegans intestine group than in the C. elegans gonad group at BLAST bit-score cutoff of either 50 or 100, indicating the intestinal expression of homologous genes tend to be maintained across nematodes. However, the number of homologous pairs detected between the two gonad groups was not different from that between the gonad and intestine groups. (0.14 MB PPT) [file pntd.0000269.s002.ppt]
