# Supplementary material for: Intestinal Transcriptomes of Nematodes: Comparison of the Parasites Ascaris suum and Haemonchus contortus with the Free-living Caenorhabditis elegans
Source: PLoS Negl Trop Dis. 2008 Aug 6;2(8):e269. doi: 10.1371/journal.pntd.0000269 (PMC2483350; doi:10.1371/journal.pntd.0000269)
Supplement: Text S1 — Supplementary Materials. (0.04 MB DOC) [file pntd.0000269.s011.doc]

**Supplementary Materials**

**Sequence Similarities Identified in the Parasite ESTs**

To provide a first overview of gene identities in the two parasites, all available EST clusters were translated and queried against three phylogenetically specific sequence groups covering all the currently available coding sequences in public databases (Figure S1). In total, 53% (*A. suu*m) and 75% (*H. contortus*) of EST clusters contained similarities to known genes in other organisms. The higher percentage for *H. contortus* was likely due to the fact that most nematode sequences currently available are generated from clade V species. The remaining clusters without similarities (47% and 25%, respectively) include novel genes that are either lineage- or species-specific.

Distributions of the identified sequence similarities to the different sequence groups were nearly identical in the two parasites (Figure S1; Figure S2). About 60% of all homologous EST clusters (i.e. EST clusters found similar to known sequences) had putative homologs in all the three sequence groups, implying that they are likely to be involved in common molecular and cellular processes conserved across metazoans. In contrast, 14-15% of the homologous EST clusters were found to contain similarities in coding sequences restricted to *Caenorhabditis spp.* and other nematodes, making them candidates for nematode-specific genes. Furthermore, small subsets of genes (31 *A. suum* and 5 *H. contortus* EST clusters) showed similarities restricted to non-nematode coding sequences, suggesting either species-specific gene acquisition in their genomes, gene-loss events or accelerated changes in other nematodes, or contaminations of host genes. Incomplete genomes and lack of representations for many nematode species could also contribute to this. This group had matches to enzymes such as cobyric acid synthase (AS15280.cl) and alpha-mannosidase (AS16547.cl), amino acid transporter (AS09071.cl), and ion transport protein (AS11163.cl). Finally, 21% or 23% *A. suum* and *H. contortus* EST clusters, respectively, were similar only to non-Caenorhabditis nematode sequences, the majority of which (> 90%) originated from parasitic nematodes. In fact, among the genes of this category, only 54 *A. suum* and 24 *H. contortus* EST clusters showed similarities to any of the ~14,000 EST sequences from *Pristionchus pacificus* and *Zeldia punctata*, the only other free-living nematodes with sequence information available (data not shown). Therefore, this group is interesting because it may contain broadly conserved genes that are important to parasitism.

**Gene Ontology Analysis on the IntFam Groups Other Than IntFam-241**

As for the IntFam-241 group, statistically enriched gene ontologies were identified for non-Intfam-241 groups (Table S6). These ontologies may include protein functions specific to different nematode lineages and species. However, the protein families were built on partial transcriptomes, some of them may become new members of the conserved “core” intestinal transcriptome containing homologous genes from all the three nematodes, when more intestinal sequences become available. This makes it difficult to identify the true lineage- or species- specific characteristics. For example, IntFam-47 (the 47 protein families containing sequences from only *A. suum* and *H. contortus*; Figure 4) had 37 genes identified as electron transporters that are likely involved in energy generation (GO:0006118) (Table S6). All of them were annotated according to their strong similarities to essential members of the canonical electron transport-coupled ATP synthesis, such as the NADH dehydrogenase subunit I or the cytochrome C oxidase subunit III (data not shown). Even though these IntFam-47 families do not currently contain *C. elegans* intestinal members, it is difficult to imagine the lack of those energy generation components in *C. elegans* intestinal cells. In fact, orthologous genes for all of them have been identified and annotated in the *C. elegans* genome ([www.wormbase.org](http://www.wormbase.org/)), suggesting this result was probably caused by the incompleteness of the intestinal transcriptome in *C. elegans*.

**Supporting Figure Legends**

**Figure S1.** Distribution of Sequence Similarities Identified in *A. suum* and *H. contortus* EST Clusters. The three phylogenetically specific sequence groups used to identify sequence similarities of the intestinal genes were: i) *Caenorhabditis spp.*, amino acid sequences from the complete genomes of *C. elegans*, *C. briggsae*, and *C. remanei*, ii) Other Nematoda, non-Caenorhabditis nematode nucleic acid sequences excluding those from either *A. suum* or *H. contortus*, when sequences from *A. suum* or *H. contortus* were queried, respectively, and iii) Non-Nematoda, non-nematode amino acid sequences from the non-redundant protein database NR. In total, 53% (5,303/9,947) *A. suu*m and 75% (3,792/5,058) *H. contortus* EST clusters contained primary sequence similarities to known genes from other species, but similar distributions of the identified matches to various species groups were observed in the two parasites.

**Figure S2.** Homologous Pairs between the Intestine and Gonad Gene Groups from *A. suum* and *C. elegans*. Significant larger number of genes in the *A. suum* intestine group had homologous counterparts in the *C. elegans* intestine group than in the *C. elegans* gonad group at BLAST bit-score cutoff of either 50 or 100, indicating the intestinal expression of homologous genes tend to be maintained across nematodes. However, the number of homologous pairs detected between the two gonad groups was not different from that between the gonad and intestine groups.
